# High resolution melting: a useful field-deployable method to measure *dhfr* and *dhps* drug resistance in both highly and lowly endemic *Plasmodium* populations

**DOI:** 10.1186/s12936-017-1811-2

**Published:** 2017-04-19

**Authors:** Yaye Dié Ndiaye, Cyrille K. Diédhiou, Amy K. Bei, Baba Dieye, Aminata Mbaye, Nasserdine Papa Mze, Rachel F. Daniels, Ibrahima M. Ndiaye, Awa B. Déme, Amy Gaye, Mouhamad Sy, Tolla Ndiaye, Aida S. Badiane, Mouhamadou Ndiaye, Zul Premji, Dyann F. Wirth, Souleymane Mboup, Donald Krogstad, Sarah K. Volkman, Ambroise D. Ahouidi, Daouda Ndiaye

**Affiliations:** 1 Laboratory of Parasitology Mycology, Aristide le Dantec Hospital, Faculty of Medicine and Pharmacy, Cheikh Anta Diop University, 5005 Dakar, Senegal; 2 Laboratory of Bacteriology and Virology, Hospital Aristide Le Dantec, 7325 Dakar, Senegal; 3 Department of Parasitology and Medical Entomology, Muhimbili University College of Health Sciences, Dar-es-Salaam, Tanzania; 4 Tulane University, New Orleans, LA USA; 5 The International Centers of Excellence for Malaria Research (ICEMR) Program, Dakar, Senegal; 6 Department of Immunology and Infectious Diseases, Harvard TH Chan School of Public Health, Boston, MA 02115 USA; 7 Department of Pathology, Aga Khan University Hospital, Nairobi, Kenya; 8 Infectious Disease Initiative, The Broad Institute of MIT and Harvard, Cambridge, MA 02142 USA; 9 School of Nursing and Health Sciences, Simmons College, Boston, MA 02115 USA

**Keywords:** *Plasmodium falciparum*, *dhfr*, *dhps*, HRM, PCR/RFLP, Senegal, Tanzania

## Abstract

**Background:**

Emergence and spread of drug resistance to every anti-malarial used to date, creates an urgent need for development of sensitive, specific and field-deployable molecular tools for detection and surveillance of validated drug resistance markers. Such tools would allow early detection of mutations in resistance loci. The aim of this study was to compare common population signatures and drug resistance marker frequencies between two populations with different levels of malaria endemicity and history of anti-malarial drug use: Tanzania and Sénégal. This was accomplished by implementing a high resolution melting assay to study molecular markers of drug resistance as compared to polymerase chain reaction–restriction fragment length polymorphism (PCR/RFLP) methodology.

**Methods:**

Fifty blood samples were collected each from a lowly malaria endemic site (Sénégal), and a highly malaria endemic site (Tanzania) from patients presenting with uncomplicated *Plasmodium falciparum* malaria at clinic. Data representing the DHFR were derived using both PCR–RFLP and HRM assay; while genotyping data representing the DHPS were evaluated in Senegal and Tanzania using HRM. Msp genotyping analysis was used to characterize the multiplicity of infection in both countries.

**Results:**

A high prevalence of samples harbouring mutant DHFR alleles was observed in both population using both genotyping techniques. HRM was better able to detect mixed alleles compared to PCR/RFLP for DHFR codon 51 in Tanzania; and only HRM was able to detect mixed infections from Senegal. A high prevalence of mutant alleles in DHFR (codons 51, 59, 108) and DHPS (codon 437) were found among samples from Sénégal while no mutations were observed at DHPS codons 540 and 581, from both countries. Overall, the frequency of samples harbouring either a single DHFR mutation (S108N) or double mutation in DHFR (C59R/S108N) was greater in Sénégal compared to Tanzania.

**Conclusion:**

Here the results demonstrate that HRM is a rapid, sensitive, and field-deployable alternative technique to PCR–RFLP genotyping that is useful in populations harbouring more than one parasite genome (polygenomic infections). In this study, a high levels of resistance polymorphisms was observed in both *dhfr* and *dhps*, among samples from Tanzania and Sénégal. A routine monitoring by molecular markers can be a way to detect emergence of resistance involving a change in the treatment policy.

## Background

*Plasmodium falciparum*, the most deadly species of *Plasmodium* parasites that infect humans, remains a public health problem with the majority of cases and deaths occurring in sub-Saharan Africa [1]. Anti-malarial drug resistance is a major public health problem that hinders the control of malaria. *P. falciparum* resistance has been observed for all anti-malarial drugs used to date, including the artemisinin derivatives, where resistance has emerged in Asia [2–4]. Continuous monitoring of the effectiveness of anti-malarial drugs both in vivo and in vitro plays a critical role in guiding treatment policy. Monitoring molecular markers of resistance is a quick and effective way to identify changes in drug resistance in real time. Malaria remains an important public health issue generally in Africa, and specifically in Sénégal and Tanzania, causing significant morbidity and mortality in infants and pregnant women [2]. In Sénégal, the epidemiological profile is characterized by a stable endemic malaria, marked by a seasonal increase, with parasite prevalence trends having declined overall from 5.9% in 2008 to 1.2% in 2014 [5]. However, malaria incidence remains elevated, especially in parts of the country where deaths attributable to malaria persist [6]. In contrast, malaria transmission in Mlandizi, Tanzania is perennial [7], with a high burden of malaria infection and clinical disease as indicated by the 678,207 reported cases of malaria in 2014 that resulted in 5368 deaths from malaria [2].

Chloroquine (CQ) was the treatment of choice against the uncomplicated malaria in both Tanzania and Sénégal for decades. However, rising rates of CQ resistance led Tanzania to change its first-line treatment from CQ to sulfadoxine–pyrimethamine (SP) in 2001 and then to artemisinin-based combination therapy (ACT) in 2006 [8]. Sénégal changed from CQ to SP-amodiaquine (AQ) in 2003 for use in seasonal malaria chemoprevention defined as the intermittent administration of full treatment courses of an anti-malarial medicine to children during the malaria season in areas of highly seasonal transmission (SMC) and then to ACT as first-line treatment of uncomplicated *P. falciparum* in 2006. SP remains in use for intermittent pregnancy treatment (IPT) in both countries [9–12].

SP is a combination of two antifolate compounds sulfadoxine that inhibits dihydropteroate synthetase (DHPS) and pyrimethamine that targets dihydrofolate reductase (DHFR). This combination acts synergistically against *P. falciparum*, and SP resistance mutations have been well documented. Mutations resulting in the following amino acid changes N51I, C59R, S108N and I164L have been identified in the *dhfr* gene associated with resistance to pyrimethamine [13–19]. Mutations resulting in the following amino acid changes S436A, A437G, K540E and A613T/S in the *dhps* locus have similarly been linked to sulfadoxine resistance [20–25]. Despite high levels of resistance to SP in many countries, this drug combination is still widely used for treatment of uncomplicated malaria, for preventing malaria in pregnant women in the context of IPT [2, 26], or in combination with artemisinin derivatives for SMC as recommended by the World Health Organization (WHO). Routine monitoring of genetic resistance mutations affecting SP efficacy is useful in determining whether the drugs should continue to be used for treatment of uncomplicated malaria or malaria pregnancy.

Different methods have been developed to evaluate the association of single nucleotide polymorphisms (SNPs) and specific phenotypes. Polymerase chain reaction (PCR) restriction fragment length polymorphism (PCR–RFLP), Taqman real-time PCR with allele-specific probes, and denaturing gradient gel electrophoresis (DGGE) are the most commonly used techniques that are suitable for these types of studies [27, 28]. PCR/RFLP is time consuming and needs specific restriction enzymes for each SNP, as well as the ability to resolve and visualize the products using gel electrophoresis. DGGE requires extensive expertise that is not always available in disease endemic settings. Furthermore, the Taqman fluorescent probes are expensive and reagents expire rapidly. High-resolution melting (HRM) analysis is a post-PCR analysis method designed to investigate variance in nucleic acid sequences [29]. Many studies have already published the accuracy, specificity and sensitivity of this technique, and its ability to detect minor alleles [30–32], and identify new genetic variants that can be confirmed by sequencing [29, 31]. HRM is a powerful analysis tool for large-scale genotyping as it is rapid, low cost and easy to deploy in the field.

The goals of this study was to: (1) compare the results of HRM to those using PCR–RFLP in the context of drug resistance marker surveillance in a malaria endemic country; and, (2) to determine the prevalence of mutations N51I, C59R, S108N in the *dhfr* gene and A437G, K540E, A581G, A613T/S in the *dhps* gene, across two malaria endemic settings with distinct frequencies of polyclonal infections (infections harbouring more than 1 parasite genome), as determined by MSP 1 and 2 genotyping.

## Methods

### Study population

This study was conducted using samples from two African countries: Sénégal and Tanzania, with distinct malaria endemicity profiles. Samples from Senegal were collected in Thiès, an urban area located 70 km from capital city of Dakar, at the Service de Lutte Antiparasitaire (SLAP) clinic. In this region, malaria is hypoendemic with average of 0–20 infective bites per person per year (0 < EIR < 20) [33].

Samples from Tanzania, were collected from the Mlandizi Health Centre in the Kibaha coastal region 40 km north–west of Dar es Salaam. In this area, malaria transmission is perennial, with peaks incidence occurring toward the end of the long (May–July) and short (December–January) rains [7, 34]. Individuals seeking treatment for uncomplicated *P. falciparum* malaria at the SLAP clinic in Thies in 2011 and the Mlandizi Health Centre in 2003–2004 were tested for malaria infection by microscopy. Patients between the ages of two and twenty who presented with only *P. falciparum* confirmed by positive blood slide were offered enrollment into this study.

These studies were approved by the Tanzanian Commission for Science and Technology (Permit No. 2003-207-CC-2003-102) together the Ethics Committee of the Ministry of Health in Sénégal (0127MSAS/DPRS/CNRES). Ethical review and approval was then provided by both the Harvard T.H. Chan School of Public Health Human Subjects Committee (P11778-101), and the Human Subjects Committee of Tulane University, New Orleans.

### Sample collection

After informed consent, blood samples from finger-pricks were collected and stored on Whatman FTA filter papers prior to treatment with SP in Tanzania (2003) and ACT (artemether–lumefantrine) in Sénégal (2011), according to the directives of the WHO and Ministry of Health in both countries, at the time of collection. Fifty samples from each country were randomly selected for DNA extraction and further genetic analyses.

### DNA extraction

Genomic DNA was extracted from filter paper using the QIAamp DNA Mini kit (Qiagen) method. The extraction protocol for filter paper samples was performed and all samples were processed in the same way. Extracted DNA was stored at −20 °C until tested by PCR–RFLP and HRM.

### Genotyping methods

#### Polymerase chain reaction restriction fragment length polymorphism (PCR–RFLP)

Sample analysis was based on the standardized polymerase chain reaction and restriction fragment length polymorphism method, as previously described [35]. After PCR amplification, 0.5 unit of site specific restriction enzymes were used to digest the PCR amplicons overnight as described previously by Jelinek et al. [35]. Positive (3D7 and Dd2) and negative non-template controls were included in all amplification and restriction digest procedures.

#### High resolution melting (HRM)

The reaction was performed on a LightScanner-32 carousel platform using primers and probes as previously described [29]. Glass capillaries were used with a 10 µl final volume. Combining both mutant allele amplification bias **(**MAAB) [29] and glass capillaries are ideal for measuring low minor allele frequencies (0.01%) in mixed genomic samples, which was one of the goals of this analysis. All PCR reactions were performed using 2.5× LightScanner master mix (Biofire), with forward primers at a final concentration of 0.05 µM, reverse primers at a final concentration of 0.2 µM (asymmetric PCR), and allele specific probes at a final concentration of 0.2 µM, and 1 µl of genomic DNA, as previously described [29]. Standard software included with the instruments was used for unlabeled probe analysis to visualize melting peaks based on different melting temperatures, indicative of different base pairs, and compared with controls to call alleles for a given assay.

#### *msp* genotyping

Block 2 of *msp1* [36] and block 3 of *msp2* [37] were amplified by nested PCR. The sequence of the primers and the protocol of PCR are described in detail by Snounou et al. [38]. Briefly, PCR was carried out in a total volume of 20 µl that contained 6 µl Gotaq (Taq DNA polymerase, dNTPs, MgCl_2_ and reaction buffer (pH 8.5), 0.05 µM of each primer and 11 µl of reagent grade water. For the first round of amplification, 1 µl of genomic DNA was added as a template and for the second round 1 µl of the PCR product from the first round was added. Reference strain 3D7 (*msp1*-K1 and *msp2*-IC); Dd2 (*msp1*-MAD20 and *msp2*-FC) and 7G8 (*msp1*-RO) were used as positive controls. Reagent grade water was used as negative control. Products were analysed based on size differences on a 2% agarose gel. The multiplicity of infection (MOI) was defined as the greater number of alleles for either *msp1* or *msp2* from a single sample.

The number of patients with more than one amplified PCR fragment within the total population is defined as the frequency of polyclonal infections. The parasite genome number was also estimated to approximate the number of distinct genotypes present in each sample. Thus, *msp* genotyping data were used to estimate genotypes per patient and after that the prevalence of each allele was determined in both countries. However, if the patient presents with a mixed infection with 4 clones, the result was called ‘undetermined’ since it could be 1 wild-type (WT) and 3 mutant (Mut); 2 WT and 2 Mut; or, 3 WT and 1 Mut.

### Statistical analysis

Analysis data was performed using Epi Info7. Fischer’s exact test was used to determine the concordance between PCR–RFLP and HRM and the z-test for two population proportions was used to compare the allele prevalence in each country. The Mann–Whitney U test was used to compare the MOI in the sample populations of each country. The test is significant if the p value is less than 0.05.

## Results

### Comparison to PCR/RFLP and HRM

Data from the *dhfr* gene corresponding to codons 51, 59 and 108 were used to compare the concordance between the PCR/RFLP and HRM assays. Previous studies have specifically compared the sensitivity and accuracy of this HRM method to the gold-standard of sequencing amplicons, and have found HRM results to be 100% correspondent [29]. However, here the goal was to assess concordance and sensitivity, in situations in which discrepancies were observed in the concordance, the method in which more alleles was detected was considered to be more sensitive. A total of 100 samples: 50 samples from Sénégal and 50 from Tanzania were genotyped using both techniques. Sénégal and Tanzania were selected for the comparison as the two countries have different frequencies of mixed infections and potentially different minor alleles and frequencies. Both techniques were performed in a laboratory in a malaria-endemic country (Sénégal) to assess their performance. In this study, a high prevalence of mutant alleles was observed at codons 51, 59 and 108, with some notable differences.

In Sénégal, a high proportion of monoallelic infections was detected using both PCR/RFLP and HRM methods, but only HRM detected a low level of mixed allelic infections. In Tanzania, a country with more polygenomic infections, HRM was better able to detect the mixed alleles among samples, and the frequency of mixed allelic infections detected by HRM were higher than those obtained by PCR/RFLP at codon 51 (p = 0.005) and 59 in Tanzania (Table 1).

**Table 1:** Percent prevalence of *dhfr* alleles at codons 51, 59 and 108 from isolates collected in Senegal and Tanzania using nested polymerase chain reaction/restriction fragment length polymerase (PCR/RFLP) and high resolution melting (HRM)

		Senegal (N: 50)			Tanzania (N: 50)		
		PCR/RFLP	HRM	p value	PCR/RFLP	HRM	p value
DHFR 51	N51	1/50 (2%)	0	1	09/50 (18%)	08/50 (16%)	1
	51I	49/50 (98%)	47/50 (94%)	0.6173	37/50 (74%)	26/50 (52%)	0.0365
	N51 + 51I	0	3/50 (6%)	0.2424	04/50 (08%)	16/50 (32%)	0.005
DHFR 59	C59	2/50 (4%)	1/50 (2%)	1	19/50 (38%)	12/50 (24%)	0.1941
	59R	48/50 (96%)	46/50 (92%)	0.6777	24/50 (48%)	22/50 (44%)	0.8411
	C59 + 59R	0	3/50 (6%)	0.2424	07/50 (14%)	16/50 (32%)	0.0558
DHFR 108	S108	2/50 (4%)	0	0.4949	07/50 (14%)	07/50 (14%)	1
	108N	48/50 (96%)	49/50 (98%)	1	35/50 (70%)	38/50 (76%)	0.6528
	S108 + 108N	0	1/50 (2%)	1	08/50 (16%)	05/50 (10%)	0.5535

*N* total number of patient

### Prevalence of mutations in Senegal and Tanzania in *dhfr/dhps* genes by HRM

Mutation analysis was successful at each codon analysed from the *dhfr* and *dhps* genes, that included the three codons (N51I, C59R and S108N) in *dhfr* and four codons (A437G, K540E, A581G and A613T/S) in *dhps*. The prevalence of mutations at each codon in monogenomic, polygenomic, and combined infections (as defined by MSP-typing) is shown in Table 2.

**Table 2:** Prevalence of mutations in *dhfr* and *dhps* in Senegal and Tanzania: monogenomic, polygenomic, and combined

Genes	Alleles	Monogenomic infections			Polygenomic infections			Combined Prevalence of mutations		
		Senegal	Tanzania	p value	Senegal	Tanzania	p value	Senegal	Tanzania	p value
DHPS 437	A437	10/23 (43.47%)	6/14 (42.86%)	0.9681	8/27 (29.62%)	12/36 (33.33%)	0.75656	18/50 (36%)	18/50 (36%)	1
	G437	12/23 (52.17%)	6/14 (42.86%)	0.58	16/27 (59.25%)	11/36 (30.56%)	0.0226	28/50 (56%)	17/50 (34%)	0.0271
	A437 + G437	1/23 (4.34%)	2/14 (14.29%)	0.28	3/27 (11.11%)	13/36 (36.11%)	0.02382	4/50 (8%)	15/50 (30%)	0.00512
DHPS 540	K540	23/23 (100%)	14/14 (100%)	≥0.05	27/27 (100%)	36/36 (100%)	≥0.05	50/50 (100%)	50/50 (100%)	≥0.05
	E540	0/23 (0%)	0/14 (0%)	≥0.05	0/27 (0%)	0/36 (0%)	≥0.05	0	0	≥0.05
	K540 + E540	0/23 (0%)	0/14 (0%)	≥0.05	0/27 (0%)	0/36 (0%)	≥0.05	0	0	≥0.05
DHPS 581	A581	23/23 (100%)	14/14 (100%)	≥0.05	27/27 (100%)	36/36 (100%)	≥0.05	50/50 (100%)	50/50 (100%)	≥0.05
	G581	0/23 (0%)	0/14 (0%)	≥0.05	0/27 (0%)	0/36 (0%)	≥0.05	0	0	≥0.05
	A581 + G581	0/23 (0%)	0/14 (0%)	≥0.05	0/27 (0%)	0/36 (0%)	≥0.05	0	0	≥0.05
DHPS 613	A613	21/23 (91.30%)	14/14 (100%)	0.25848	26/27 (96.29%)	36/36 (100%)	≥0.05	46/50 (92%)	49/50 (98%)	0.16758
	T/S613	1/23 (3.33%)	0/14 (0%)	0.42952	0/27 (0%)	0/36 (0%)	≥0.05	2/50 (4%)	0	0.15272
	A613 + T/S613	1/23 (3.33%)	0/14 (0%)	0.42952	1/27 (3.7%)	0/36 (0%)	0.24604	2/50 (4%)	1/50 (2%)	0.5552
DHFR 51	N51	0/23 (0%)	4/14 (28.57%)	0.00672	0/27 (0%)	4/36 (11.12%)	0.07346	0	09/50 (18%)	0.00168
	51I	20/23 (86.95%)	4/14 (28.57%)	0.0003	27/27 (100%)	22/36 (61.12%)	0.00024	47/50 (94%)	37/50 (74%)	0.00634
	N51 + 51I	3/23 (13.04%)	6/14 (42.86%)	0.04036	0/27 (0%)	10/36 (27.78%)	0.00278	3/50 (6%)	04/50 (08%)	0.69654
DHFR 59	C59	0/23 (0%)	4/14 (22.57%)	0.00672	1/27 (3.70%)	8/36 (22.22%)	0.03752	1/50 (2%)	19/50 (38%)	0
	59R	20/23 (86.95%)	4/14 (22.57%)	0.0003	26/27 (96.29%)	18/36 (50%)	0.0001	46/50 (92%)	24/50 (48%)	0
	C59 + 59R	3/23 (13.04%)	6/14 (42.86%)	0.04036	0/27 (0%)	10/36 (27.78%)	0.0088	3/50 (6%)	07/50 (14%)	0.18352
DHFR 108	S108	0/23 (0%)	5/14 (35.71%)	0.00208	0/27 (0%)	2/36 (5.55%)	0.21498	0	07/50 (14%)	0.00614
	N108	22/23 (95.65%)	9/14 (64.29%)	0.01208	27/27 (100%)	29/36 (80.56%)	0.0151	49/50 (98%)	35/50 (70%)	0.00014
	S108 + N108	1/23 (4.35%)	0/14 (0%)	0.42952	0/27 (0%)	5/36 (13.89%)	0.04338	1/50 (2%)	08/50 (16%)	0.01428

Msp-1 and Msp-2 typing data were combined with drug resistance allele typing to determine the prevalence of mutations at each codon in monogenomic, polygenomic. The z-test for 2 population proportions was used to compare the allele prevalence in each country. The test is significant if the p value is less than 0.05

In this study, mutant alleles at codons A581G and K540E in *dhps* gene were not found in among the samples analysed from Tanzania and Sénégal, and all samples tested carried the wild type alleles A581 and K540, respectively. The analyses showed that, monoclonal infections were more common in Sénégal, with a high frequency of single mutant alleles at codons 437 in *dhps* and codons 51 (p = 0.0003), 59 (p = 0.0003) and 108 (p = 0.012) in *dhfr*. However, the vast majority of infections are polyclonal in Tanzania, and the frequency of mixed allele calls was also higher compared to Sénégal just as mixed allele was more represented in monogenomic infections (codons 51 and 59 (p = 0.04)) and polygenomic infections (codons 437 (p = 0.02) and 51 (p = 0.002), codons 59 (p = 0.008) and 108 (p = 0.04) (Table 2).

Typing resistance alleles by either PCR–RFLP or HRM yields a result for all parasite genomes in a given patient sample. In an attempt to tease out the number of resistant “genomes” in the patient population, drug resistant allele typing was combined with MSP typing data to determine the number of wild-type or mutant genomes present at each locus (Table 3). Overall, there were more polygenomic infections in Tanzania (72%) compared to Sénégal (54%) (Table 3), although the difference was not statistically significant. However, when considering the average multiplicity of infection for each site, Tanzania had a significantly higher MOI compared to Sénégal (MOI Tanzania = 2.6, compared to MOI Sénégal = 1.56; p = 0.011). The overall results remained unchanged whether the data was analysed as the resistance profile for the sample population (Table 2) or weighted based on the number of parasite genomes (Table 3).

**Table 3:** Prevalence of mutations in *dhfr* and *dhps* in Senegal and Tanzania when accounting for number of parasite genomes per sample

	Alleles	Parasite genome		
		Senegal	Tanzania	p value
DHPS 437	A437	27/80 (33.75%)	42/103 (40.78%)	0.9729
	G437	45/80 (56.25%)	39/103 (37.86%)	0.0131
	Undetermined	8/80 (10%)	22/103 (21.36%)	
DHPS 540	K540	80/80 (100%)	103/103 (100%)	>0.05
	E540	0/80 (0%)	0/103 (0%)	>0.05
	Undetermined	0/80 (0%)	0/103 (0%)	
DHPS 581	A581	80/80 (100%)	103/103 (100%)	>0.05
	G581	0/80 (0%)	0/103 (0%)	>0.05
	Undetermined	0/80 (0%)	0/103 (0%)	
DHPS 613	A613	75/80 (3.75%)	100/103 (97.09%)	0.2713
	T/S613	2/80 (2.5%)	0/103 (0%)	0.1074
	Undetermined	3/80 (3.75%)	3/103 (2.91%)	
DHFR 51	N51	0/80 (0%)	21/103 (20.39%)	0
	51I	77/80 (96.25%)	67/103 (65.05%)	0
	Undetermined	3/80 (3.75%)	15/103 (14.56%)	
DHFR 59	C59	1/80 (1.25%)	32/103 (31.07%)	0
	59R	76/80 (95%)	56/103 (54.37%)	0
	Undetermined	3/80 (3.75%)	15/103 (14.56%)	
DHFR 108	S108	0/80 (0%)	16/103 (15.5%)	0.0002
	N108	79/80 (98.75%)	87/103 (84.5%)	0.0096
	Undetermined	1/80 (1.25%)	0/103 (0%)	
	Polyclonal infections	27/50 (54%)	36/50 (72%)	0.0628
	Multiplicity of infection	78/50 (1.56)	103/50 (2.06)	0.011

The number of parasite genotypes per patient was calculated to estimate the wild-type and mutant allele frequencies in mixed infections. Undetermined represents samples in which the number of genotypes cannot be precisely classified due to uncertainty (for example, if there are 4 genomes, the call could be 1 WT and 3 Mut, 2 WT and 2 Mut, or 3 WT and 1 Mut)

When combining the mutant alleles into haplotypes, the single mutation S108 N (p = 0.01) and double mutation C59R/S108N (p = 0.005) in the *dhfr* gene were higher in Senegal than in Tanzania but the triple N51I/C59R/S108N mutation on *dhfr* gene and the quadruple N51I/C59R/S108N *dhfr* and A437G *dhps* gene mutation were more represented in Tanzania, albeit not significantly different (Table 4). The quintuple mutation was not observed in either site.

**Table 4:** Prevalence of single, double, triple, quadruple and quintuple mutation in Tanzania and Senegal

	Single mutation (%)	Double mutation (%)	Triple mutation (%)	Quadruple mutation (%)	Quintuple mutation (%)
Senegal	20	22	44	52	0
Tanzania	2	2	48	58	0
p value	0.01	0.005	0.84	0.7	0

Mutant alleles from *dhfr*, *dhps* genes were combined to make the single mutation (S108N), double mutation (*dhfr* C59R/S108N), triple mutation (*dhfr* N51I/C59R/S108 N), quadruple mutation (*dhfr* N51I/C59R/S108N dhps A437G) and quintuple mutation (N51I/C59R/S108N *dhfr* and A437G/K540E *dhps*)

## Discussion

This study assessed the accuracy of HRM in comparison with PCR–RFLP for detecting infections of *P. falciparum* in two areas Mlandizi, Tanzania and Thiès, Sénégal two regions with variable endemicity and transmission intensity.

HRM analysis is comparable to PCR–RFLP for classifying SNPs; however, PCR–RFLP is laborious, time consuming, and requires a specific restriction enzyme for each SNP. This method also requires the separation of PCR products on a gel, which often takes hours to perform and increases the risk of contamination, making it difficult to genotype a large number of samples. Furthermore, interpretation of the digestion profiles can be subjective in cases of suboptimal digestion, low DNA yields, faint PCR products. Here, the results demonstrate that even when performed in a malaria-endemic laboratory setting, HRM is a rapid, accurate, powerful, economic, and a “closed-tube” mutation typing method that detects sequence variation within the PCR products, and can detect minor alleles in a mixed genotype population of parasite DNA. As described by previous studies, HRM can identify known and novel polymorphisms, detect multiple genotypes, and is both sensitive and specific [29, 30, 38–41]. This study applied the HRM technology to type polymorphisms in mixed genotype infections in two African countries.

In Tanzania, more mixed genotypes were identified by HRM than PCR/RFLP at codon 51 (p = 0.005), 59 and 108. In Sénégal, a country with fewer polygenomic infections, no mixed infections was observed by PCR/RFLP, however several were detected by HRM, although the small number resulted in non-significant p-values (Table 1). These results demonstrate that HRM is more sensitive than PCR/RFLP and can easily detect mixed alleles. Since PCR–RFLP may not detect clones which are at low frequency in a mixed population, due to the qualitative nature of the assay, a minor allele could easily pass unnoticed. In contrast, HRM detected mixed infections at a higher frequency in both populations, suggesting that the technology of HRM to detect minor subpopulations is more sensitive than PCR–RFLP. While in countries like Senegal with few polygenomic infections and a low multiplicity of infection, there may not be a significant difference in the techniques; whereas, the improved sensitivity and ability to detect minor alleles is more pronounced in sample populations such as Tanzania with a high prevalence of polygenomic infections and a higher multiplicity of infection. This makes HRM a more attractive and accurate method for typing samples from both countries, but especially in countries like Tanzania, which are characterized by a high frequency of mixed infections. Furthermore, the ability to detect rare, low-frequency drug resistance alleles is likely important for surveillance of these markers as drug pressure is applied and likely to select for such variants.

As HRM was the most sensitive method evaluated, it was used exclusively for determining the genotype of *dhfr* and *dhps* genes to look at the drug resistance profiles in both countries. The frequency of mutant alleles at codon 437 in *dhps* gene and at codons 51, 59 and 108 on *dhfr* gene associated with in vivo and in vitro to SP resistance [23, 42] was higher in Sénégal and Tanzania (Table 2). These high frequencies of mutation were observed in a study conducted in Dakar, Senegal [43] and in Tanzania [44]. The presence of mutations at codons 540, 581 on *dhps* gene was not detected in either country.

In both countries, the high prevalence of mutations in *dhfr* and *dhps* could be explained by the use of SP as a second line treatment for malaria in Senegal and first line in Tanzania at the time of sample collection. In Sénégal, SP has been used since 2003 in combination with amodiaquine for use as SMC for children; whereas, in Tanzania, SP was introduced in 2001 as first line treatment for uncomplicated malaria but removed in 2006 due to the high level of resistance observed in vivo and in vitro. SP remains the mainstay drug regime for intermittent preventative treatment of pregnant women (IPTp) in both countries. It is very possible that the continued use of SP may favour stepwise selection of mutations in these areas, contributing to the high prevalence of mutant alleles observed in this study. The mutation A437G in the *dhps* gene and N51I, C59R and S108N in *dhfr* gene were more prevalent in Senegal than in Tanzania (Tables 2 and 3), which is interesting given that there has been longer term SP pressure in Tanzania compared to Sénégal. It has been observed in some studies that SP resistance emerges more rapidly in low-transmission compared to high-transmission areas [45], and this is consistent with the results of this study.

One potential confounder to the more frequent resistant alleles in Sénégal compared to Tanzania is the difference in the MOI between the two sites. As many infections in Tanzania are polygenomic and contain a high MOI, it is possible that the number of mutant alleles circulating in the population is underestimated as both PCR-RFLP and HRM can classify alleles as mutant or wild-type, but cannot determine the number of alleles of each (just the total population profile: all wild-type, all mutant, or mixed). To address this challenge, *msp*-*1* and *msp*-*2* typing data was combined with drug resistance allele typing to determine the number of wild-type or mutant parasite genomes (Table 3). When accounting for the frequency on a parasite genome basis (rather than a per human basis), the results do not significantly change as mutant alleles are still higher in Sénégal than Tanzania.

Often, studies report combinations of mutations in both *dhfr* and *dhps* as a way to compare with WHO guidelines for continued SP use. When combining mutant alleles, the single mutation (*dhfr* S108N) and the double mutation (*dhfr* C59R/S108N) was more represented in Sénégal than in Tanzania with p = 0.01 and p = 0.005 respectively (Table 4). Triple and quadruple mutations were not significantly different between the two sites, although they were high for both sample sets. Encouragingly, the quintuple mutation N51I/C59R/S108N *dhfr* and A437G/K540E *dhps* gene, which is strongly associated with in vivo and in vitro SP resistance in East and Southern Africa [46, 47] was not observed, consistent with findings from previous studies in Sénégal by Ndiaye et al. [48, 49] and Wurtz et al. [43]. However, a recent study conducted in Sénégal found a single sample with the quintuple mutation [50], resulting in an overall population prevalence of 1.1%. In light of this result, continued and constant monitoring of drug resistance molecular markers is essential.

## Conclusion

These results demonstrate the enhanced sensitivity of HRM assays to detect minor mutant alleles compared to PCR/RFLP strategies in samples derived from two endemic countries with different levels of malaria burden. Notably, Tanzania exhibited a higher MOI compared to Sénégal; and DHFR mutations were more common among samples from Senegal, as compared to Tanzania. Based upon the mutant allele frequencies and the absence of quintuple mutations predictive for SP resistance these populations, these data indicate that SP likely remains efficacious for IPTp and SMC per WHO recommendations. However, as very recently a sample with the quintuple mutation was observed in Sénégal, continued and constant monitoring of drug resistance molecular markers by robust, sensitive, and field-deployable methods like HRM is a high priority.

## Abbreviations

CQ: chloroquine
SP: sulfadoxine–pyrimethamine
ACT: artemisinin-based combination therapy
AQ: amodiaquine
SMC: seasonal malaria chemoprevention
IPT: intermittent pregnancy treatment
DHPS: dihydropteroate synthetase
DHFR: dihydrofolate reductase
WHO: World Health Organization
SNPs: single nucleotide polymorphisms
PCR–RFLP: polymerase chain reaction–restriction fragment length polymorphism
DGGE: denaturing gradient gel electrophoresis
HRM: high-resolution melting
MSP: mérozoide surface protein
SLAP: Service de Lutte Antiparasitaire
EIR: entomological inoculation rates
DNA: deoxyribonucleic acid
dNTPs: désoxyribonucléotides triphosphates
Mgcl_2_: chlorure de magnesium
pH: potentiel hydrogene
MOI: multiplicity of infection
WT: wild type
Mut: mutant
p: p value
